# Metanephric Adenoma: A Case Report of a Rare Epithelial Renal Tumor

**DOI:** 10.7759/cureus.58545

**Published:** 2024-04-18

**Authors:** Gregory Szlyk, Lydia G Capicotto

**Affiliations:** 1 Urology, Mary Washington Hospital, Fredericksburg, USA; 2 Urology, Urology Associates of Fredericksburg, Fredericksburg, USA; 3 Medical School, Edward Via College of Osteopathic Medicine, Blacksburg, USA

**Keywords:** immunohistochemical, percutaneous biopsy, abdominal imaging, renal neoplasm, robot-assisted

## Abstract

Metanephric adenoma (MA) is a rare embryonal epithelial tumor that is often diagnosed incidentally. A definitive diagnosis can only be made postoperatively based on the pathological findings. This case report discusses the rare occurrence of a metanephric adenoma, the symptoms it can present with, and the diagnosis, treatment, and immunological staining needed to differentiate metanephric adenoma from other types of renal tumors. In this study, a 37-year-old female presented to the emergency room for vague right lower quadrant pain (RLQ) and underwent imaging that showed a lesion on the lower pole of the left kidney. A subsequent biopsy of the lesion showed a low-grade renal epithelial neoplasm favoring metanephric adenoma. The patient successfully underwent a left partial nephrectomy to remove the tumor, which required no further treatment after resection. Due to the rarity of the tumor, it requires immunohistology to differentiate from other renal tumors such as Wilm’s tumor and renal cell carcinoma. This case report aims to recognize proper workup, diagnosis, and treatment to achieve a positive outcome in the setting of this rare tumor.

## Introduction

Metanephric adenoma (MA) is a rare embryonal epithelial tumor of the kidney with an excellent prognosis. It accounts for 0.2% of all adult renal tumors. It is often found and diagnosed incidentally but can present with non-specific symptoms such as flank pain, hematuria, palpable mass, and polycythemia. It was first described by Bove et al. in 1979, and several hundred have been reported in English literature [1, 2]. It was first believed that MA was a variety of Wilms tumor or papillary renal cell carcinoma due to the imaging and microscopic appearance, but it has since been described as its own entity.

Preoperatively, MA does not have any features to distinguish between different renal tumors. A definitive diagnosis can only be made postoperatively via histopathological findings and staining. Metanephric adenoma commonly affects middle-aged females and males in a 2:1 ratio [3, 4]. This study describes the case of a 37-year-old female who was diagnosed with a metanephric adenoma that was found incidentally on imaging.

## Case presentation

**Figure 1:**
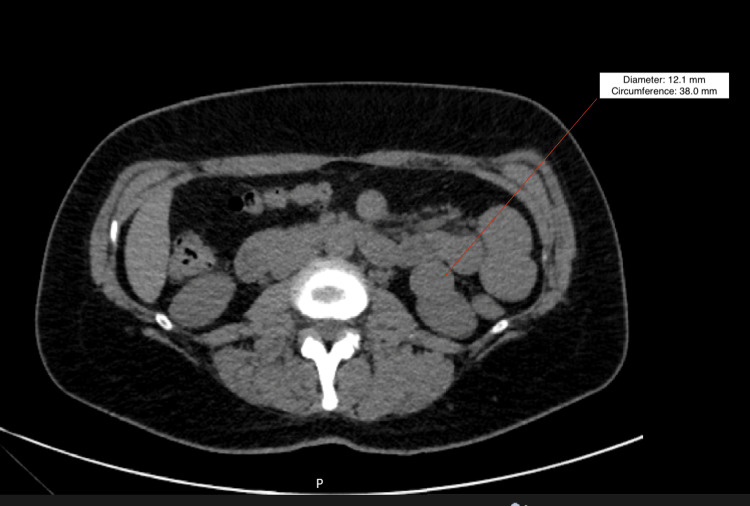
A CT scan of the abdomen and pelvis with and without contrast The red arrow indicates the lesion of the left kidney, measuring 2.5 cm x 2.2 cm.

A 37-year-old Caucasian female with a medical history of hypertension, polycystic ovary syndrome (PCOS), and endometriosis presented to the emergency room with a complaint of right low quadrant (RLQ) pain. She stated that the pain was different from her pain due to PCOS and endometriosis. Her vital signs were stable, and her physical exam was positive for RLQ tenderness without rebound or guarding. Initial laboratory workup showed an unremarkable complete blood count (CBC), comprehensive metabolic panel (CMP), urinalysis, and a negative serum human chorionic gonadotropin (hCG). A CT scan of the abdomen and pelvis with IV contrast and a transvaginal and pelvic ultrasound were performed in the ED. Figure 1 presents the CT image. Incidentally, imaging showed a left renal lesion on CT and normal findings on pelvic ultrasound. A diagnosis for the RLQ pain was not determined. She denied symptoms of fever, chills, changes in urination, weight change, and hematuria. Her RLQ pain was relieved while in the emergency room. She was recommended to follow up with her primary care physician for a workup of the incidental left renal lesion. 

**Figure 2:**
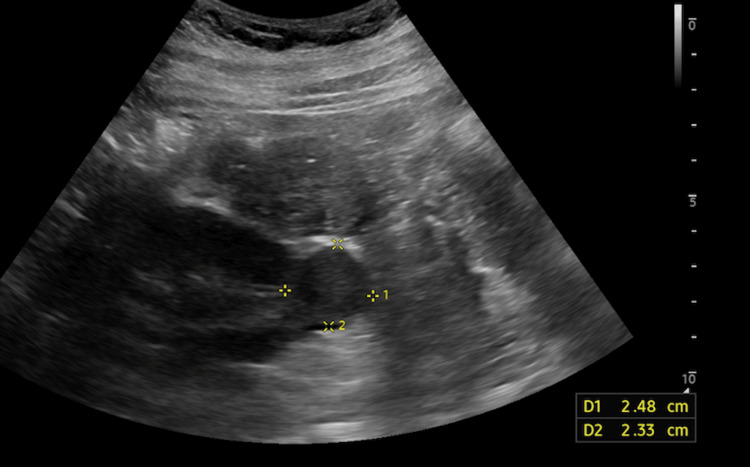
Transabdominal grayscale and duplex sonography of the retroperitoneum

Shortly following the ED visit, she followed up with her primary care physician, who recommended a referral to urology to investigate and work up the unknown kidney mass. Urology recommended a dedicated renal ultrasound and a CT scan of the abdomen and pelvis with and without IV contrast to be performed as they did not have access to previous imaging from the ED visit. The renal ultrasound images are presented in Figure 2. 

**Figure 3:**
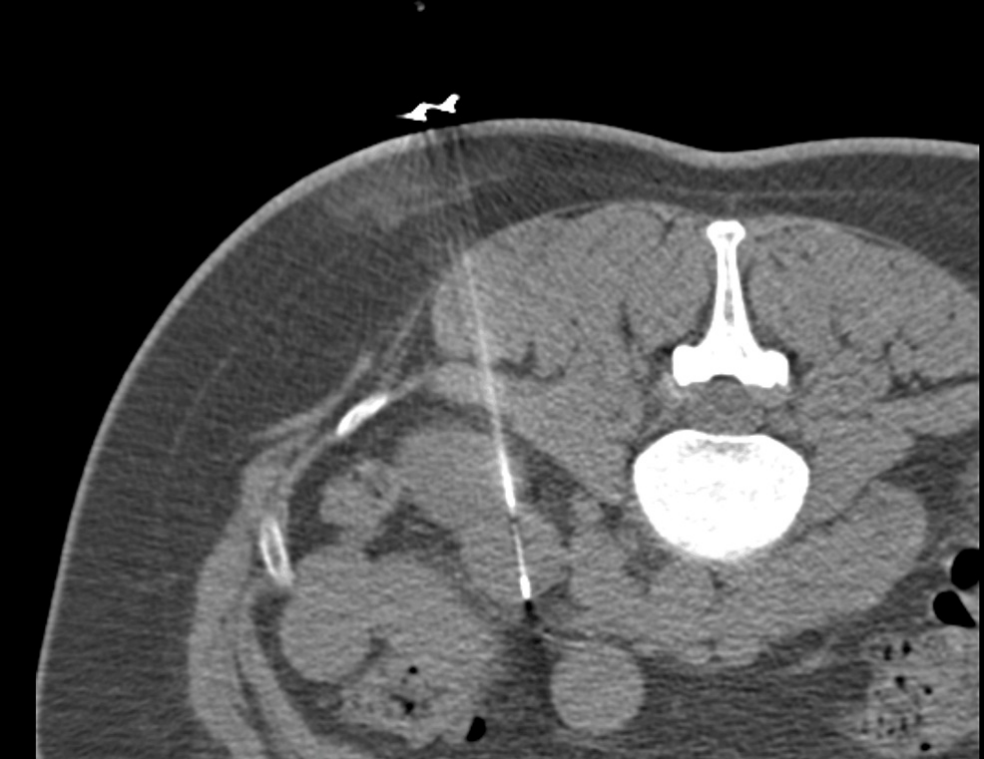
A CT-guided core needle biopsy of the left renal lesion

She then underwent imaging, in which the ultrasound showed a lesion in the lower pole of the left kidney measuring 2.5 x 2.3 x 2.6 cm, whose differential diagnoses included renal cell carcinoma or oncocytoma. The CT showed an enhancing 2.5 x 2.2 cm solid exophytic mass on the anterior inferior pole of the left kidney and recommended histologic sampling. Due to the imaging results, the patient proceeded with a recommended CT-guided biopsy of the mass (Figure 3). The CT-guided biopsy was successful without any complications. Initial pathology showed a low-grade renal epithelial tumor composed of tubular structures lined by scant cytoplasm. These pathology results favored metanephric adenoma.

**Figure 4:**
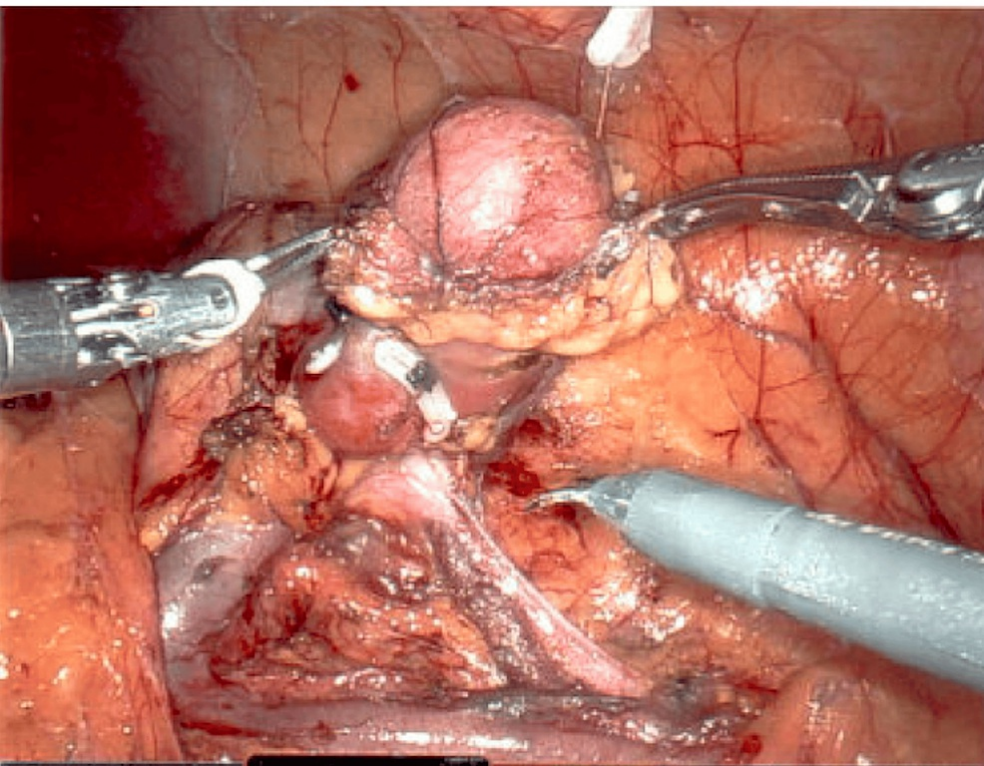
An intraoperative photo of the gross tumor (inferior view of the lower pole of the left kidney)

It was recommended for the mass to be removed. If excision cannot be performed, close clinical follow-up is warranted. The patient proceeded with the removal of the mass. She underwent a successful robotic-assisted left partial nephrectomy. A tan-yellow to tan-brown, well-encapsulated mass measuring 2.5 x 5 x 2.5 cm was removed with clear margins (Figure 4). The final pathology showed a tumor composed of tubular papillary architecture with small cuboidal cells with mild cytologic atypia and no mitotic activity or necrosis. The tumor showed Ki-67 proliferation and positivity for WT1 and BRAFV600 markers. It was negative for CK7, S-100, and CD117 markers. No further treatment was needed after resection, and the patient had no postoperative complications.

## Discussion

The diagnosis and management of renal tumors are vital. The etiology and risk factors of metanephric adenomas are widely unknown. More than half of reported cases are asymptomatic, but common symptoms that have been reported are flank pain, hematuria, palpable mass, and polycythemia [4,5]. A variety of symptoms have been reported to initiate a workup for the patient. A metanephric adenoma is otherwise most often found incidentally on imaging. Metanephric adenoma appears to have an indolent course with slow growth over the years. Imaging of the tumor, including both CT and ultrasound, can show a well-defined, encapsulated mass on any portion of the kidney [5]. A metanephric adenoma is hyperdense in the surrounding renal parenchyma. Calcifications, hemorrhagic areas, and necrosis can be seen on imaging [5]. The imaging characteristics of MA lack specificity and thus require further investigation. A preoperative biopsy can be done for a preemptive diagnosis, which was performed in this case, but it does not come without risks. The complications of percutaneous biopsy include post-procedure hemorrhage, seen in 1.2% of cases, and perirenal soft tissue infection, seen in 0.2% of cases [6]. Arteriovenous fistulas can occur in up to 14% of cases but are clinically silent and will spontaneously resolve [6]. An official diagnosis can only be made postoperatively once the tumor has been resected.

Macroscopically, MAs tend to be gray, yellowish, well-encapsulated solid masses. Histopathologically, tumor cells contain renal epithelial or stromal cells with uniformly small, ovoid nucleoli [4]. There is minimal to no mitosis seen [4]. A distinct interface between the tumor and surrounding renal parenchyma can be visualized on microscopy. If renal mass resembles the histology of MA, immunohistology should be performed. Immunohistologically, the MA stain is positive for WT1, vimentin, PAX8, and CD57. Metanephric adenoma stains are negative for alpha-methylacyl-CoA racemase (AMACR) and cytokeratin 7 [7]. This staining pattern distinguishes MA from papillary renal cell carcinoma, which stains positive for AMACR and cytokeratin 7. Wilms tumor can be differentiated from MA in staining as it is positive for WT1 and PAX8 and negative for vimentin and CD57 [8]. Genetically, the presence of the BRAF V600E mutation plays a role in the pathogenesis of MA, as seen in the reported patient [7]. 

Although most believed to be a benign tumor, reports of lymph node metastasis have been reported [1, 9]. It is recommended to surgically remove the tumor in a timely manner. The nephron-sparing technique, known as a partial nephrectomy, is recommended when technically feasible. Ablative techniques, such as radiofrequency or cryotherapy, may be considered in patients who are poor surgical candidates. The data on ablation techniques for metanephric adenomas are minimal, as the tumor is not able to undergo histological typing after the procedure. If excisions are not performed, close clinical follow-up is advised, but no recommendations or guidelines have been made for appropriate surveillance [10].

## Conclusions

The purpose of this case study is to highlight the rarity of the tumor and its presentation. It is important to differentiate metanephric adenoma from other renal tumors. Diagnosis can only be obtained through tissue sampling, as imaging can be non-specific. A biopsy can be performed preoperatively with a recommendation from the physician, but not without risks. A surgical removal of the mass is recommended for diagnosis and treatment. The timely workup and treatment of this patient provided good outcomes. 
